# What is the effect of bodily illusions on corticomotoneuronal excitability? A systematic review

**DOI:** 10.1371/journal.pone.0219754

**Published:** 2019-08-15

**Authors:** Alex Dilena, Gabrielle Todd, Carolyn Berryman, Ebonie Rio, Tasha R. Stanton

**Affiliations:** 1 BodyinMind Research Group, School of Health Sciences, Division of Health Sciences, University of South Australia, Adelaide, South Australia, Australia; 2 School of Pharmacy and Medical Sciences, Division of Health Sciences, University of South Australia, Adelaide, South Australia, Australia; 3 Neuromotor Plasticity and Development (NeuroPAD) Research Group, Robinson Research Institute, School of Medicine, University of Adelaide, Adelaide, South Australia, Australia; 4 La Trobe Sport and Exercise Medicine Research Centre, School of Allied Health, La Trobe University, Melbourne, Victoria, Australia; 5 Neuroscience Research Australia, Randwick, New South Wales, Australia; Universite Laval, CANADA

## Abstract

**Background:**

This systematic review aimed to summarise and critically appraise the evidence for the effect of bodily illusions on corticomotoneuronal excitability.

**Methods:**

Five databases were searched, with two independent reviewers completing study inclusion, risk of bias, transcranial magnetic stimulation (TMS) reporting quality, and data extraction. Included studies evaluated the effect of an illusion that altered perception of the body (and/or its movement) on excitability of motor circuitry in healthy, adult, human participants. Studies were required to: use TMS to measure excitability and/or inhibition; report quantitative outcomes (e.g., motor evoked potentials); compare the illusion to a control or active comparison condition; evaluate that an illusion had occurred (e.g., measured illusion strength/presence).

**Results:**

Of 2,257 studies identified, 11 studies (14 experiments) were included, evaluating kinaesthetic illusions (n = 5), a rubber hand illusion (RHI) paradigm (n = 5), and a missing limb illusion (n = 1). Kinaesthetic illusions (induced via vision/tendon vibration) increased corticomotoneuronal excitability. Conflicting effects were found for traditional, visuotactile RHIs of a static hand. However, embodying a hand and then observing it move (“self-action”) resulted in decreased corticomotoneuronal excitability and increased silent period duration (a measure of Gamma-Aminobutynic acid [GABA]_B_-mediated intracortical inhibition in motor cortex), with the opposite occurring (increased excitability, decreased inhibition) when the fake hand was not embodied prior to observing movement (“other-action”). Visuomotor illusions manipulating agency had conflicting results, but in the lower risk study, illusory agency over movement resulted in a relative decrease in corticomotoneuronal excitability. Last, an illusion of a missing limb reduced corticomotoneuronal excitability.

**Conclusion:**

While evidence for the effect of bodily illusions on corticomotoneuronal excitability was limited (only 14 experiments) and had a high risk of bias, kinaesthetic illusions and illusions of embodying a hand (and seeing it move), had consistent effects. Future investigations into the role of embodiment and the illusion strength on corticomotoneuronal excitability and inhibition are warranted.

## Introduction

Illusions that alter one’s perception of the body by manipulating sensory or motor input are a useful way to explore the relationship between body perception and the neurophysiological regulation of our body [1]. There is growing evidence that bodily illusions can induce neural changes in healthy [2] and in clinical populations [3]. For example, vision of a longer arm that appears to be your own (first-person perspective) induces an illusion that dynamically updates the neural representation of touch for the arm in the primary somatosensory cortex [4]. Further, magnifying vision of the hand results in an updated motor representation in the primary motor cortex (M1) [5]. Given that many clinical conditions, such as post-stroke and pathological limb pain, have impaired motor function with evidence of neural changes to motor areas [6, 7], the effect of illusions on the motor cortex and, particularly, their potential to alter excitability is an emerging area of research.

**Fig 1:**
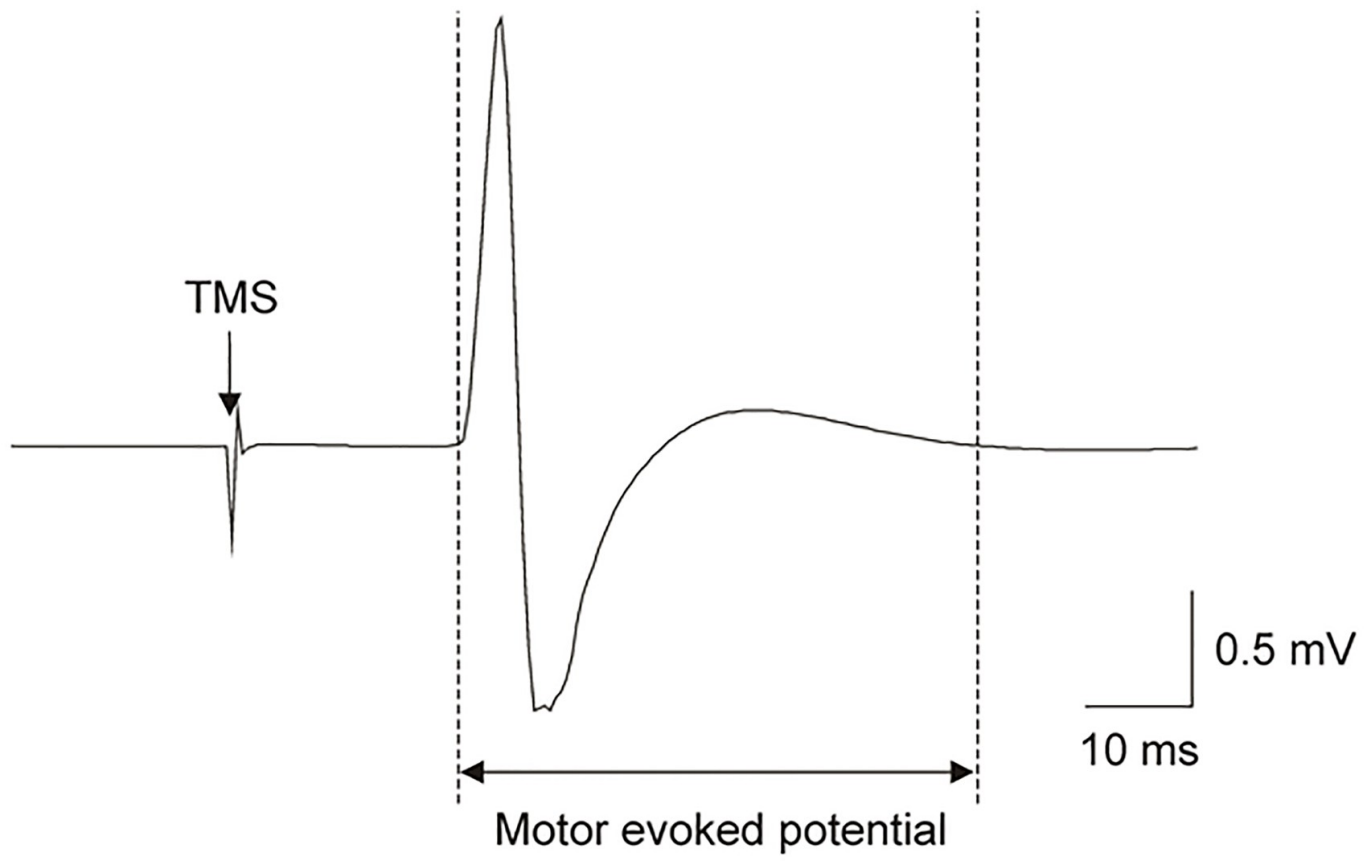
Illustrative motor evoked potential. Single-subject data showing an averaged trace of electromyographic (EMG) activity in the first dorsal interosseous (FDI) muscle during relaxation. The arrow shows the timing of single-pulse transcranial magnetic stimulation (TMS) delivered over the contralateral FDI area of the primary motor cortex. The average trace was calculated from 12 pulses delivered at ~0.2 Hz. The EMG response to TMS is called a motor evoked potential (MEP). The size (area or peak-to-peak amplitude) of the MEP reflects corticomotoneuronal excitability. TMS, transcranial magnetic stimulation; mV, millivolts; ms, milliseconds.

The excitability of the pathway between the motor cortex and the muscle (termed corticomotoneuronal excitability) can be measured using transcranial magnetic stimulation (TMS). A magnetic pulse is delivered to M1 through the scalp and the stimulus-evoked response in the target muscle is measured using surface electromyography (EMG), with the amplitude of this response termed the motor evoked potential (MEP; Fig 1) [8]. Research in people post-stroke or with chronic pain has shown that altering corticomotoneuronal excitability via non-invasive brain stimulation, such as repetitive TMS (rTMS), can have important clinical benefits. For example, high frequency repetitive stimulation (>3 Hz) rTMS to the affected motor cortex (aiming to increase corticomotoneuronal excitability) or low frequency repetitive stimulation (≤1 Hz) to the unaffected motor cortex (aiming to decrease corticomotoneuronal excitability) have been shown to enhance motor recovery post-stroke [9, 10]. Similarly, rTMS has been shown to improve the analgesic efficacy of traditional medical treatments for chronic pain (versus sham rTMS) [11]. Given that contraindications to non-invasive brain stimulation exist (e.g., epilepsy, metallic hardware, some central nervous system active drugs) [12] and that clinically relevant treatment effects may require high dosages [13, 14], exploring other ways to induce changes in corticomotoneuronal excitability for neurorehabilitation is relevant. Indeed, the potential use of body illusions in neurorehabilitation has been highlighted as an important research avenue [15], particularly given recent findings showing promising positive effects of body illusions on pain [16]. Here we aim to take the first step in understanding the neurophysiological effects of body illusions on the motor cortex by exploring the evidence for illusions to alter corticomotoneuronal excitability in healthy participants. While illusions of movement are intuitively relevant to the motor cortex and its function, given that action requires perceptual and sensory input about the size, shape, and location of the body part [17], illusions that manipulate body perception are also important to consider.

The most common bodily illusions involve the use of unisensory (e.g., vision) or multisensory input (e.g., vision + touch) to induce an altered perception of the body part (e.g., size or location) or of its movement. For example, kinaesthetic illusions use unisensory input to induce a feeling that the body part is moving when it is not. Kinaesthetic illusions can be induced via visual stimuli–i.e., by viewing limb movement (of another person or via mirrored reflection) in a first-person perspective. The mirror neuron system [18] is hypothesised to play a role in the perception of illusory movement, given its activation in humans when observing movement of others [19]. Kinaesthetic illusions can also be induced via tendon vibration. Tendon vibration excites type I and II muscle spindle afferents, which, during normal movement, activate in response to muscle stretch to provide proprioceptive input of limb position [20]. Thus, when tendon vibration is applied to an immobilised limb (e.g., to an elbow flexor tendon), muscle spindles from the agonist muscle (i.e., the biceps) signal that the muscle is being stretched, creating an illusion of arm movement in the direction of the antagonist muscle action (i.e., of elbow extension).

In contrast, the rubber hand illusion (RHI) is induced by synchronous visuotactile input–stroking both a rubber hand (placed visibly and in an anatomically plausible position in front of the participant) and the real, hidden hand [21] to manipulate the sense of body ownership (see [22] for a comprehensive review). It is proposed that this synchronous stroking of both the real and rubber hand allows the sensory input to be interpreted as coming from a common event [23] and that embodiment of the rubber hand may also be dependent on incoming sensory input being integrated into numerous pre-existing internal body maps, (e.g., comparing the visual appearance, the postural alignment, etc., of rubber hand to pre-existing body models) [24]. While numerous brain regions have been proposed to contribute to the sense of ownership, including the parietal cortex, premotor cortex, intraparietal sulcus, and insula (for full review see [22, 25]), recent evidence suggests that the motor system also plays a critical role in body ownership. For example, the premotor cortex integrates multisensory input from vision and touch [26] and its neural activity (shown using functional magnetic resonance imaging; fMRI) reflects the feeling of ownership induced by the RHI [1]. Lesions to the premotor cortex can result in somatoparaphrenia [27], a condition in which an otherwise healthy limb is disowned (no longer feels like it belongs to you). In addition, M1 may also play a role in ownership. TMS to the motor area of the hand induces embodiment of a virtual hand (when visual input of thumb movement is congruent to the TMS-induced involuntary muscle contraction) [28]. Moreover, the absence of limb movement (people with complete unilateral hemiplegia) results in heightened RHI ownership experiences for the affected limb (vs unaffected limb and healthy controls) [29].

The RHI can also be induced using synchronous visuomotor input–moving your real hand and seeing a rubber hand move at the same time to influence body ownership and also the sense of agency (i.e. the sense that the actions we perform are our own). It is proposed that the brain has an internal prediction model whereby an efference copy of a motor command is created and is compared to actual sensory input resulting from the movement [30, 31]. If the efference copy matches the afferent input (proprioceptive input from the moving hand, visual input of the moving object), then the movement is perceived as being self-caused and agency arises. This theory suggests likely involvement of both sensory and motor areas [32]. Indeed, neural correlates of agency include the motor system (supplementary motor areas, the ventral premotor cortex, and the cerebellum), as well as the multimodal association cortices (e.g., posterior parietal cortex, dorsolateral prefrontal cortex, and posterior segment of the superior temporal sulcus and the insula) [30].

There is substantial evidence that body-relevant sensory information (e.g. vision of the body) influences corticomotoneuronal excitability. For example, viewing another’s movement (i.e., action observation) has been consistently found to elicit increased corticomotoneuronal excitability [19, 33, 34]. What is less clear, however, is whether similar changes in corticomotoneuronal excitability occur during illusions–is merely having a perception of movement (without actual movement occurring) sufficient to induce changes in motor cortex function? Such knowledge has potential relevance for conditions in which movement is not possible (e.g., post-stroke). There is evidence that imagining performing a movement (i.e., motor imagery) modulates corticomotoneuronal excitability [35, 36]. However, this represents a self-generated perception (i.e., perception of body movement *without* additional sensory stimuli [37]). Given that some people find motor imagery difficult [38], particularly in clinical conditions such as stroke [39], with studies showing minimal corticomotoneuronal excitability changes for those with low imagery ability [40], understanding the effects of illusory movement (i.e., perception of body movement *induced by* additional sensory stimuli) remains relevant.

There is preliminary evidence that bodily illusions may have effects on corticomotoneuronal excitability. For example, studies have found that embodying a rubber hand (i.e., the RHI) decreases corticomotoneuronal excitability, demonstrated by a reduction in MEP amplitude [41]. The effect may also be dependent upon the type of illusion: visual kinaesthetic illusions have been shown to increase corticomotoneuronal excitability (i.e., increased MEP amplitude) [42]. Despite this promising preliminary work, a comprehensive understanding of the effects of bodily illusions on corticomotoneuronal excitability has yet to emerge. To date, there has been no attempt to review nor to critically evaluate the available evidence. Thus, the aim of this systematic review was to summarise and critically appraise the evidence for the effect of bodily illusions on corticomotoneuronal excitability in a healthy human population. Such findings will provide an important basis for understanding how perceptual alterations of the body interact with motor function in the healthy nervous system.

## Materials and methods

This review conformed to guidelines from the Preferred Reporting Items for Systematic Reviews and Meta-analyses (PRISMA) statement [43]. We conducted this review following an *a priori* protocol (available upon request from T.R.S.). In all stages, two independent reviewers (A.D. and T.R.S.) completed the tasks. Any disagreements were discussed between the reviewers and if unable to come to a consensus, a third reviewer was consulted (G.T.).

### Data sources

A systematic search was performed in 5 databases (Medline via OvidSP, PubMed, PsychINFO, Embase via OvidSP, Web of Science) from inception up to January 2018 to identify studies evaluating the effect of bodily illusions on corticomotoneuronal excitability. Key words and subject headings relevant to illusions, corticomotoneuronal excitability, and TMS were used in the search strategies and were updated for each database. See S1 File for the Medline search strategy. Additionally, the reference lists of all studies for which full text was retrieved were hand searched for potentially relevant studies. Results from the database searches were exported to Endnote (X8, Clarivate Analytics, Philadelphia, USA) where duplicates were removed and then the final search results were uploaded to Covidence (www.covidence.org/) for online review.

### Study eligibility criteria

Studies were included if they evaluated the effect of an illusion that altered perception of the body (and/or body movement) by measuring corticomotoneuronal (i.e., from motor cortex to muscle) or corticocortical (i.e., within the cortex itself) excitability during the illusion using single- or paired-pulse TMS in healthy, adult (>18 years of age), human participants. Specifically, studies were required to have evaluated at least one of the following outcomes: corticomotoneuronal excitability (single-pulse MEP area or amplitude); Gamma-Aminobutynic acid [GABA]_B_-mediated intracortical inhibition (silent period duration of >100 milliseconds [ms]); GABA_A_-mediated short-interval intracortical inhibition (SICI; paired-pulse MEP amplitude at 2–3 ms interstimulus interval); intracortical facilitation (ICF; paired-pulse MEP amplitude at 10–12 ms interstimulus interval). To be included, studies were required to have compared the effect of bodily illusions on excitability measures to that of a control condition/group (i.e. no illusion; sham illusion), or to have made an active comparison (e.g., one type of illusion versus another type of illusion). Studies were also required to have evaluated that a body illusion actually occurred (e.g., included a relevant measure of illusion strength or illusion presence). Studies in which both clinical samples and healthy control samples were recruited were considered eligible if the healthy control data were provided separately.

Studies were excluded if there was no control or comparison condition, if adjunctive interventions known to influence corticomotoneuronal excitability were used (e.g., if rTMS, an intervention known to modulate corticomotoneuronal excitability, was used concurrently with bodily illusions), if GABA_B_-mediated intracortical inhibition (i.e. silent period duration) was measured at <100 ms (i.e., reflecting only spinal excitability, not corticomotoneuronal excitability), or if the illusion did not alter the perception of a body part (e.g. altering the environment to create an illusion versus altering the body part). All animal studies were excluded. Case studies (defined as studies where the unit of analysis was one participant) were also excluded.

### Defining bodily illusions

This study used an established definition of a bodily illusion [16]. That is, a bodily illusion was defined as “a phenomenon in which an external stimulus is interpreted by the neural system in such a way that the resultant perception of the body is significantly different from reality. This may include alterations to the size/shape, location, movement, or ownership (e.g., rubber hand illusion) of the painful body part. This includes illusions of movement of the body part and/or illusory existence of an amputated body part [16] (Pg 517).”

### Study inclusion

Two independent reviewers screened potential studies by title and abstract to remove obviously irrelevant studies. Following this, full texts of potentially eligible studies were retrieved and formally assessed by the independent reviewers for eligibility (on Covidence) using the above criteria. The eligibility criteria were piloted by the two reviewers on three studies to ensure comprehensiveness, understanding, and agreement.

### Risk of bias assessment and TMS reporting quality

The same two independent reviewers assessed the risk of bias of included studies using a custom-designed, piloted tool, which evaluated the presence of selection bias, detection bias, blinding, statistical bias, reporting bias, performance bias, attrition bias, and other forms of relevant bias (e.g. control for confounding variables). See S2 File for the full version of this tool. The Strengthening the Reporting of Observational Studies in Epidemiology (STROBE) guidelines [44] were used to inform risk of bias assessment for cross-sectional, repeated measures, and observational study designs. This modified risk of bias tool has been used in past reviews to evaluate risk of bias for experimental study design level evidence [16]. For randomised controlled trials (RCTs), additional questions on allocation concealment and adequate sequence generation were included as suggested by the Cochrane Collaboration [45].

The established TMS quality checklist was used to evaluate the quality of reporting of TMS methods [46]. Studies were considered high quality if they met all the TMS checklist features. For single pulse paradigms, the maximum score is 26. For paired pulse paradigms, the maximum score is 30.

### Data extraction

A customised, piloted data extraction form was used by the same two independent reviewers to extract the following data: participant demographics (i.e. number, gender, age, handedness), study design (within- versus between-group comparison), type of illusion (i.e. kinaesthetic, rubber hand, virtual reality), control condition/group used (i.e. no illusion, sham or active illusion condition/group), assessment method of body perception change. The TMS testing parameters that were extracted included: the cortical testing site(s), including the cerebral hemisphere for TMS application; the target muscle for electromyography measurement for MEPs (e.g. FDI or Abductor Digiti Minimi [ADM]); the number of stimuli provided/MEPs measured; and the stimulus intensity (% of active/resting motor threshold/other). For paired-pulse paradigms, the interstimulus interval (e.g. 2 or 3 ms) and the stimulus intensity of the conditioning and the test pulse was also extracted.

For all TMS outcomes, measures of central tendency (mean, median) and variability (standard deviation, interquartile range) were extracted for all provided time points, including pre-intervention, during intervention, and post-intervention, for each group/condition. If insufficient data were provided, authors were contacted a maximum of three times to retrieve this data.

### Data synthesis and analysis

Included studies were grouped according to type of bodily illusion evaluated, the comparison condition/group used, and the type of excitability outcome evaluated. Illusion categories were defined based on the potential of the illusion methodology to impact corticomotoneuronal excitability, not based on the percept altered. For example, the RHI can be induced using visuotactile input (an individual sees the rubber hand being touched as their real hand is touched) or visuomotor input (an individual sees the rubber hand move as their real hand moves). A moving vs stationary hand will clearly have a differential impact on corticomotoneuronal excitability, thus precluding direct comparison of these illusions, despite evaluating similar perceptual constructs. Additionally, kinaesthetic illusions induce a feeling of movement without actual movement occurring. This perception of movement can be induced through tendon vibration or through watching movement in a first-person perspective. Similarly, both types of kinaesthetic illusions may have different impacts on corticomotoneuronal excitability.

Effect sizes were calculated for each study comparison when possible. For within-subject analyses, Cohen’s d for dependent samples was used and based on body illusion and corticomotoneuronal excitability data from our lab (in preparation), a within-subject correlation of 0.6 was used. For between subject analyses, Cohen’s d for independent samples was calculated. For significant main effects or interactions in between group analyses, the F-statistic (and group size) were used to calculate Cohen’s d. A meta-analysis, using a random effects inverse variance approach (Revman 5.2 software) to calculate the pooled mean difference (and 95% confidence interval), was considered when two or more studies evaluated a similar illusion, compared with a similar control group/condition, and evaluated the same TMS outcome. The Grading of Recommendations, Assessments, Development and Evaluations (GRADE) assessment of level of evidence was considered if meta-analyses could be performed. The GRADE analysis considers features such as risk of bias, consistency, precision, directness, and publication bias. However, rating of the feature ‘consistency’ requires consideration of heterogeneity statistics from meta-analyses, therefore if meta-analyses are not possible, the only possible rating of level of evidence is “very low” and such conclusions can be drawn without the scale application.

## Results and discussion

**Fig 2:**
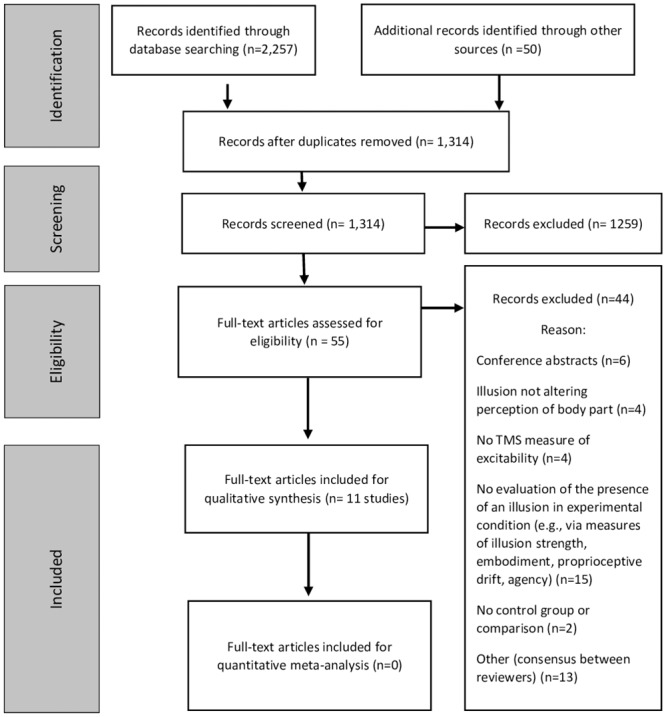
Flow diagram of the identification, screening and inclusion process.

The search resulted in 2,257 citations and hand searching retrieved a further 50 potentially relevant studies (total n = 2307). After title and abstract screening, 2252 studies were clearly ineligible resulting in retrieval of full text for 55 studies. After formal eligibility assessment of full text studies, 11 studies (14 experiments) were included in this review (Fig 2). Data were received from authors for three studies [42, 47, 48] to allow for effect size calculation. Due to study heterogeneity, meta-analyses could not be performed for any comparison. Given this, the GRADE level of evidence assessment was not performed and this reflects that the current level of evidence is very low.

### Study design

Experimental study designs were used in all included studies (i.e., no RCTs; only cross-sectional), with 10 experiments using a within-subject study design and two using a between-subject comparison. One study had a combination of within and between group data, but for analyses purposes, these data were combined and a between group analysis (one-way analysis of variance [ANOVA]) was performed [42]. Eleven experiments used randomisation of condition or group. That is, for within-subject study designs all participants completed all conditions in a randomised order and for between-subject study designs, participants were randomly allocated into one of two (or three) groups.

### Risk of bias

All included studies had a high risk of bias. Only one study reported an *a priori* sample size calculation [41]. Blinding of researchers (performing TMS analysis) was reported in only one study [49]. Blinding of participants was reported in 2 of the 13 experiments. Overall, 11 experiments adequately described the illusion, allowing for future replication. Only 6 experiments reported all results. Appropriate measures to detect changes in corticomotoneuronal excitability were used by all studies (Table 1).

**Table 1:** Risk of bias of included studies and TMS reporting quality summary scores.

Study	Selection Bias	Randomis-ation completed?	TMS method	Subject selection	Blinding of Participants	Blinding of assessors	Reporting Bias	Performance Bias (for illusion)	Confounding Variables	Statistical Methods	Attrition Bias	TMS reporting quality
**Kinaesthetic Illusions—induced by vision**												
Azoyama 2012	×	×	✓	**?**	**?**	×	✓	✓	**?**	×	**?**	8
Kaneko 2007 exp1	**?**	×	✓	**?**	**?**	×	×	✓	✓	×	✓	13
Kaneko 2007 exp2	**?**	**?**	✓	**?**	×	×	×	✓	✓	×	✓	13
Nojima 2015 exp1	×	✓	✓	**?**	✓	×	**?**	✓	**?**	×	✓	*16
Nojima 2015 exp3	×	✓	✓	**?**	✓	×	**?**	✓	**?**	×	✓	*16
**Kinaesthetic Illusions—induced by tendon vibration**												
Mancheva 2017	×	✓	✓	×	**?**	×	×	**?**	**?**	×	**?**	12
Naito 2002	×	✓	✓	**?**	**?**	×	**?**	**?**	✓	×	×	16
**Visuotactile RHI**												
della Gatta 2016	**?**	✓	✓	✓	**?**	×	✓	✓	**?**	✓	✓	15
Schütz-Bosbach 2006	**?**	✓	✓	×	**?**	×	×	✓	**?**	×	✓	13
Schütz-Bosbach 2009	**?**	✓	✓	✓	**?**	✓	✓	✓	✓	×	✓	*20
**Visuomotor RHI**												
Weiss 2014	**?**	✓	✓	✓	**?**	×	✓	✓	✓	**?**	✓	21
Karabanov 2017	×	✓	✓	**?**	**?**	×	✓	✓	×	×	**?**	19
**Missing limb illusions**												
Kilteni 2016	**?**	✓	✓	✓	×	×	✓	✓	✓	×	**?**	19

Exp, experiment; RHI, rubber hand illusion; ?, unclear; ✓, have reported and low risk; ×, have not reported and high risk; N/A, not applicable. Maximum TMS quality score of 26 (single pulse paradigms) or 30 (paired pulse paradigms).

*Paired pulse paradigm.

### Transcranial magnetic stimulation reporting quality

All studies used single-pulse TMS, with two studies also performing paired-pulse TMS [49, 50]. The mean reporting quality was 15.5 (±3.7), suggesting a low to moderate quality. Primary limitations in reporting quality related to the lack of confirmation of medication use, any medical conditions, history of specific repetitive motor activity in participants, and descriptions of the prior motor activity of the muscle to be tested.

### Effect of bodily illusions on corticomotoneuronal excitability

Four studies evaluated kinaesthetic illusions, five evaluated a RHI paradigm, and one used a missing limb illusion to investigate the effects of bodily illusions on corticomotoneuronal excitability. Of note, no mirror illusion studies were eligible for inclusion because confirmation of the presence of an illusion occurring (versus no illusion occurring) in experimental and control conditions was not evaluated. A summary of the included studies is presented in Table 2.

**Table 2:** Methodological details and results of included studies.

Type of illusion and study design	Comparison/Control	TMS details/parameters	Perceptual Measure	Summary of Results	Primary results (corticomotoneuronal excitability—illusion)
**Kinaesthetic illusions–induced by vision**					
Azoyama et al 2012, within-subject repeated measures (n = 10) [47]	Experimental conditions: Rest, DF illusion, PF illusion	Single-pulse TMS over the right motor area; stimulus intensity: 105%, 115%, and 125% of RMT–MEP amplitude; EMG: Left TA and Soleus	VAS to evaluate subjective perception of illusion	One-way RM ANOVA: Main effect of condition on TA MEP amplitude (105%: p = 0.003; 115%: p = 0.003; 125%, p = 0.025). Post hoc: ↑ TA MEP amplitude for DF illusion vs rest (all intensities) and PF illusion (105% and 115%)	↑ corticomotoneuronal excitability (TA)
	Illusion: seeing a video of the left ankle dorsiflexing and plantar flexing in 1PP; left ankle/foot stationary	Measured at the maximal DF & the maximal PF phase of the viewed moving foot.		No main effect of condition on Soleus MEP amplitude (105%: p = 0.25, 115%, p = 0.056; 125%: p = 0.43).	No change in corticomotoneuronal excitability (soleus)
				↑ Perceptual ratings for illusion conditions (i.e., participants felt like their ankle/foot was moving): DF illusion > PF illusion (p = 0.008)	
				No significant correlations between TA MEP amplitude and VAS ratings.	
Kaneko, Yasojima & Kizuka 2007, Experiment 1 within-subject repeated measures and between subjects.	Experimental conditions: Group A (n = 10): Resting, illusion, and non-illusion (control) conditions	Single-pulse TMS over the right motor area; stimulus intensity: 0.5–1 mV—MEP amplitude; EMG: Left FDI and left ADM (control).	VAS to evaluate subjective perception of illusion	One-way ANOVA: Main effect of condition on FDI MEP amplitude (p<0.001). Post hoc: ↑ MEP amplitude for FDI in illusion condition vs resting and sham (p<0.05)	↑ corticomotoneuronal excitability
Analysed as between groups via one way ANOVA (Resting, n = 20; Illusion, n = 20, non-illusion, n = 10; sham, n = 10) [42]*	Group B (n = 10): Resting, illusion, and sham (control) conditions	Delivered at mid-range of visual finger movement when vision matched actual finger position (illusion)		No effect of condition on MEP amplitude for ADM (p = 0.33)	
	Illusion: seeing video of a left index finger abducting in 1PP; left index finger held in 30 degrees of abduction	Measured during each condition		↑ Perceptual ratings for illusion condition (i.e., participants felt like their finger was moving)	
Kaneko, Yasojima & Kizuka 2007, Experiment 2 within-subject repeated measures (n = 6) [42]	Experimental conditions: Resting, index-abd (illusion)	Single-pulse TMS over the right motor area;stimulus intensity: 0.5–1 mV—MEP amplitude; EMG: Left FDI (illusion) and left ADM (control)	VAS to evaluate subjective perception of illusion	One-way RM ANOVA: Main effect of condition on FDI MEP amplitude (p<0.0029). Post-hoc: ↑ MEP amplitude for FDI in illusion condition vs all other conditions (p<0.05)	↑ corticomotoneuronal excitability
	Control conditions: index-add and little-abd, little-add (i.e., ADM)	Delivered at mid-range of visual finger movement when vision matched actual finger position (illusion).		No effect of condition on MEP amplitude for ADM (p = 0.091)	
	Illusion as above; 5^th^ finger also held in abduction	Measured during each condition		↑ Perceptual ratings for illusion condition (i.e., participants felt like their finger was moving)	
Nojima et al. 2015 Experiment 1, between-subject (n = 19) [50]	Groups: Illusion (Action observation; n = 10):Watching a ball rotation task on a LCD monitor (first-person perspective)	Single-pulse TMS over the right motor area; stimulus intensity: 1 mV—MEP amplitude; EMG: Left FDI	VAS to evaluate vividness of illusory sensation (scores not reported)	2 x 2 ANOVA: Time (pre-post) x Group (illusion, static) interaction: p = 0.023 Post-hoc tests:↑ MEP amplitude in the Illusion post-intervention (p = 0.04), but not in the Static observation group (p-value not reported)	↑ corticomotoneuronal excitability
	Control (Static observation; n = 9): Watching a still image of a left hand holding 2 balls in a LCD monitor	Paired-pulse TMS over the right motor area, conditioned MEP amplitude at 3ms (SICI) & 12ms (ICF) Conditioning stimulus		SICI–no significant results (p-value not reported)	No change in GABA_A_-mediated SICI
	Left hand undergoing all conditions	Measured pre- and post-condition		↑ ICF post-intervention in the Illusion group post-intervention (p = 0.01) but not in the Static observation group (p-value not reported)	↑ short-interval ICF
Nojima et al. 2015 Experiment 3, within-subject repeated measures (n = 10) [50]	Experimental conditions (left hand tested): Illusion (Action Observation) Watching a ball rotation task on a LCD monitor (first-person perspective)	Single-pulse TMS over the right motor area; stimulus intensity: 1 mV—MEP amplitude; EMG: Left FDI	VAS to evaluate vividness of illusory sensation (scores not reported)	One-way RM ANOVA: Main effect of condition for MEP amplitude (p = 0.006) Post-hoc: ↑ MEP amplitude in the illusion condition vs rest condition (p = 0.005)	↑ corticomotoneuronal excitability
	Action Observation-3rd: watching a ball rotation task from a third-person perspective	Paired-pulse TMS over the right motor area, conditioned MEP amplitude at 3ms (SICI) & 12ms (ICF) Conditioning stimulus		One-way RM ANOVA: No significant main effect of condition for SICI (p-value not reported).	No change in GABA_A_-mediated SICI
	Static Observation: as above	Measured during each condition		One-way RM ANOVA: Main effect of condition for ICF (p = 0.047) Post-hoc: ↑ ICF in illusion condition vs rest condition (p = 0.026)	↑ short-interval ICF
	Rest: No vision, measured at baseline and post-conditions			No other conditions differed in post-hoc testing.	
**Kinaesthetic illusions–induced by tendon vibration**					
Mancheva et al. 2017, within-subject repeated measures (n = 14; 1 did not experience the illusion) [51]	3 vision conditions: Open eyes (eyes open but preventing vision of the hand) Closed eyes Watching the vibrated wrist	Single-pulse TMS over the left motor area; stimulus intensity 120% of RMT- MEP amplitude	Self-report of perception of illusory movement (yes/no)	One-way RM ANOVA (only post-hoc reported): Low amplitude vibration (control): ↑ FCR MEP area in all conditions vs rest (p<0.05) ↓ECR MEP for closing eyes condition vs rest (p = 0.027)	
	2 vibration conditions, applied to FCR (80 Hz): Low amplitude vibration (0.5mm) High amplitude vibration (1–1.5mm)	EMG: Right FCR (muscle corresponding to the tendon being vibrated) and ECR (muscle corresponding to illusory movement)		High amplitude vibration (illusion): ↑ FCR MEP area in all conditions vs rest (p<0.05) ↑ ECR MEP area only during illusion (open eyes, no vision of hand) vs rest (p = 0.023); illusion (closed eyes) vs rest did not differ (p-values not reported)	↑ corticomotoneuronal excitability
	Illusion conditions: High amplitude vibration with Open eyes (but no vision of hand) or Closed eyes induces illusory wrist extension	Measured at rest (baseline), during each condition, and after each high amplitude vibration condition (inverse illusion; see text for results)		No illusion reported during low amplitude vibration (control). All participants reported illusory wrist extension in both illusion conditions.	
Naito, Roland & Ehrsson 2002, within-subject repeated measures (n = 8; n = 6 tested right and left hand and used for analysis) [52]	6 conditions total 3 vibration conditions: ECU tendon vibration (83 Hz) ECU tendon vibration (12.5 Hz; control) No vibration (control)	Single-pulse TMS over the left or right motor area; stimulus intensity 100% of RMT—MEP amplitude; EMG: non-vibrated FCU (left and right)	Self-report of perception of illusory movement (yes/no);	Condition (6) x Side (left, right) RM ANOVA: Illusion transfer: ↑ MEP amplitude of non-vibrated FCU only in illusion condition (Hands contacted;83 Hz) vs other conditions (p<0.001); illusion equally strong regardless of which side tested (p-values not reported)	↑ corticomotoneuronal excitability
	2 hand conditions: Contacted: palm of hand placed on the dorsum of other hand, vibration applied to ECU tendon of top hand; induces bilateral illusory wrist flexion. Separated	Measured during each condition	Replication of movement while measuring angular velocity	↓ MEP amplitude in ECU of non-vibrated hand in Illusion condition (Hands Contacted; 83 Hz) as illusion occurred; opposite to FCU (tendon x time: p<0.001)	
	Illusion: 83 Hz vibration, contacted			Confirmed experience of illusion transfer in the Contacted condition (83 Hz) (vs other conditions)	
**Visuotactile RHI**					
della Gatta et al. 2016 Experiment 1 (main), within-subject repeated measures (n = 26; 2 did not experience the illusion, so n = 24 for analysis) [41]	Illusion condition: Synchronous stroking (induces ownership over rubber hand)	Single-pulse TMS over the left motor area; stimulus intensity: 110% of RMT- MEP amplitude; EMG: Right FDI	Proprioceptive Drift	Non-parametric ANOVA: Main effect of condition (p = 0.01) Post-hoc: ↓ MEP amplitude in the illusion (synchronous) condition vs asynchronous (p = 0.0009) and baseline (p = 0.0002) conditions.	↓ corticomotoneuronal excitability
	Control condition: Asynchronous stroking	Measured at baseline and during each condition	Embodiment Questionnaire	No difference between asynchronous and baseline in MEP amplitude (p = 0.86)	
	All conditions performed on the right hand		(how much the rubber hand feels like it is your own)	Wilcoxin signed rank test: ↑ Proprioceptive drift towards rubber hand in synchronous vs asynchronous condition (p = 0.0105, d = 0.58) ↑ Embodiment in synchronous vs asynchronous (p = 0.000018, d = 3.88)	
della Gatta et al. 2016 Experiment 2 (control), within-subject repeated measures (n = 26; 6 did not experience the illusion, so n = 20 for analysis) [41]	Illusion condition: Synchronous stroking (induces ownership over rubber hand and disownership of real hand)	Single-pulse TMS over the right motor area; stimulus intensity: 110% of RMT- MEP amplitude; EMG: Left FDI	Proprioceptive Drift	One-way RM ANOVA: No effect of condition on MEP amplitude (p = 0.41)	No changes in corticomotoneuronal excitability for the respective hemisphere of the hand not undergoing the illusion (control)
	Control condition: Asynchronous stroking	*Note: testing corticomoto-neuronal excitability for the hand *not undergoing RHI*	Embodiment Questionnaire	t-tests: ↑ Proprioceptive drift towards rubber hand in synchronous vs asynchronous condition (p = 0.00004, d = 1.18	
	Conditions performed on the right hand	Measured at baseline and during each condition	Disembodiment Questionnaire (how much it feels like you have lost your real hand)	↑ Embodiment in synchronous vs asynchronous (p = 0.000001, d = 2.1) ↑ Disembodiment of real hand in synchronous vs asynchronous condition (p = 0.0012, d = 0.96)	
Schutz-Bosbach et al. 2006, within-subject repeated measures (n = 14) [48]	Illusion condition: Synchronous stroking, then observing movement of the embodied (experimenter’s) hand (“self-action”)	Single-pulse TMS over the left motor area; stimulus intensity;105% of RMT- MEP amplitude; EMG: Right FDI and ADM	Proprioceptive Drift	2 (Stroking) x 2 (Movement) ANOVA: No main effects, but significant interaction for FDI EMP amplitude (p = 0.023)	
	Control conditions: Ʌ Synchronous stroking, no hand movement (static; traditional RHI) Asynchronous stroking, hand movement (“other-action”) Asynchronous stroking, no hand movement (static; traditional RHI control)	Conditions were performed with and without TMS, the latter acting as catch trials to reduce influences of anticipation	Embodiment Questionnaire (how much the experimenter’s hand feels like it is your own)	Posthoc: Effect of illusion: ↓ FDI MEP amplitude in illusion (“self action) vs synchronous static condition (no p-values reported) Effect of vision of “other-action”: ↑ FDI MEP amplitude in asynchronous other-action vs asynchronous static (no-p-values reported	↓ corticomotoneuronal excitability during illusion (“self-action”); relative to ↑ excitability during “other-action”
	Conditions performed on the right hand	TMS pulses provided at a random interval after the start of the trial		Effect of traditional RHI: Not formally evaluated. FDI MEP amplitude static hand synchronous (traditional RHI) > than asynchronous static condition (d = 0.15)	† Unknown whether there is a change in corticomotoneuronal excitability for the traditional, static RHI
				No change in MEP amplitude for ADM (p = 0.98)	
				↑ Proprioceptive drift towards experimenter’s hand in synchronous vs asynchronous conditions (in induction phase (p<0.05) ↑ Embodiment ratings in synchronous vs asynchronous conditions (p<0.03)	
Schutz-Bosbach et al. 2009, within-subject repeated measures, 2x2 factorial design (n = 15) [49]	Illusion condition: Synchronous stroking, then observing movement of the embodied (experimenter’s) hand (“self-action”)	Single-pulse TMS over the left motor area; stimulus intensity: 130% of AMT—MEP amplitude and silent period;	Proprioceptive Drift	2 (Stroking) x 2 (Movement) ANOVA: No main effects on SP duration but significant interaction (p = 0.003)	
	Control conditions: Ʌ Synchronous stroking, no hand movement (static; traditional RHI) Asynchronous stroking, hand movement (“other-action”) Asynchronous stroking, no hand movement (static; traditional RHI control)	EMG: Right FDI (tonic contraction of FDI of 20% maximal).	Embodiment Questionnaire (as above)	Post hoc: Effect of illusion: ↑ SP duration in illusion (“self-action”) vs synchronous static (p = 0.03, d = 0.39) and asynchronous movement (“other-action”; p = 0.002, d = 1.12). Effect of vision of “other-action”: ↓ SP duration in asynchronous movement (“other-action”) vs asynchronous static hand (p = 0.019, d = 0.66)	↑ GABA_B_-mediated intracortical inhibition during illusion (“self-action”); relative to ↓ inhibition during “other-action”.
	Also used a baseline control condition	Conditions were performed with and without TMS, the latter as catch trials to ↓ influence of anticipation		Effect of traditional RHI: No difference in SP duration for synchronous static vs asynchronous static hand condition (p = 0.09, d = 0.14)	† No change in GABA_B_-mediated intracortical inhibition during traditional, static RHI
	Conditions performed on the right hand	TMS pulses provided at a random interval after the start of the trial (but after observation of hand movement)		Comparisons to baseline: ↑ SP duration in illusion (“self-action”) and synchronous static hand versus baseline (p = 0.009 and p = 0.042); asynchronous conditions did not differ (p>0.12).	
				No significant difference in MEP amplitude between conditions (no main effects or interactions: p>0.19).	No change in corticomotoneuronal excitability
				↑ Proprioceptive drift towards experimenter’s hand in synchronous vs asynchronous conditions (p = 0.043) ↑ embodiment ratings in synchronous vs asynchronous conditions, (p<0.05)	
**Visuomotor RHI**					
Karabanov et al. 2017, within-subject repeated measures (n = 7) [53]	Illusion: Agency and ownership (synchronous movement; fake hand spatially aligned with real hand)	Single-pulse TMS over the left motor area; stimulus intensity: 100% of RMT—MEP amplitude; EMG: Left FDI	Proprioceptive Drift	RM ANOVA: No difference in MEP amplitude between conditions (p = 0.11)	No changes in corticomotoneuronal excitability
	Control Conditions: 1) No agency, no ownership (asynchronous movement; fake hand not spatially aligned with real hand)	Measured during each condition; delivered during finger adduction (FDI relaxed)	Embodiment Questionnaire (perceived agency/perceived ownership items)	↑ Proprioceptive drift for illusion condition vs other conditions: p = 0.051	
	2) Agency, no ownership (synchronous movement; fake hand not spatially aligned with real hand)			↑ Ratings for agency questions vs control questions for illusion condition (p<0.001) and for Control #2 (p<0.001). Results for Control #1 not reported.	
	Left index finger movement (abduction and adduction) in all conditions.			↑ Ratings for ownership questions vs control questions for Illusion condition (p = 0.02) and for Control #2 (agency, no ownership; p = 0.04), but not Control #1 (no agency, no ownership; p = 0.47).	
Weiss et al. 2014, within-subject repeated measures (n = 29) [54]	Illusion: Synchronous visual input (visually seen movement corresponded to actual left index finger movement; induces a sense of agency)	Single-pulse TMS over the left motor area; stimulus intensity: 110% of RMT- MEP amplitude; EMG: Right FDI and ADM (control muscle)	VAS to evaluate agency (perceived control over movement)	One-way RM ANOVA (delay: 0, 100, 200, 300ms): Main effect of delay for FDI MEP ratios (p<0.001), but not ADM MEP ratios (p = 0.69)	
	Control: Asynchronous visual input (delayed visually seen movement, i.e., 100, 200, 300 ms)	Measured during each trial; time-locked with a random jitter to either the end of observed or actual movement (each 50% of trials)	Correspondence judgements (perceived delay: yes or no)	Posthoc: Relative↓ in FDI MEP amplitude during illusion (synchronous visual input; 0ms) vs asynchronous control (100ms; p = 0.017) ↑ MEP amplitude with ↑ delay in asynchronous visual input condition (300 ms > 200 ms; p = 0.042)	↑ corticomotoneuronal excitability with asynchronous delay; relative ↓ corticomotoneuronal excitability (illusion)
	Right hand tested in all conditions.			2 (delay: 100 vs 200 ms) x 2 (yes vs no correspondence) RM ANOVA: Main effect of correspondence (p = 0.034): ↓MEP amplitude for trials judged as corresponding vs non-corresponding	
				↓ VAS scores representing increased agency for illusion (synchronous visual input) vs control (asynchronous) conditions (p<0.001)	
**Missing limb illusions**					
Kilteni et al. 2016, between subject (n = 40) [55]	Groups: Amputation illusion group: A full body avatar with a missing right limb	Single-pulse TMS over the left (experimental) and right (control) motor area; stimulus intensity: 120% of RMT—MEP amplitude.	Embodiment Questionnaire with amputation specific questions	2 (group) x 2 (side) x 2 (time) ANOVA FDI: Only main effect of side (right hand > left; p<0.001) ECU: Group x time x hemisphere interaction: p = 0.053	
	Control group: Full body avatar (no missing right limb)	EMG: Right FDI and ECU (illusion), Left FDI and ECU (control)	(e.g., I felt as if part of my right arm was missing)	Exploratory post-hoc analysis: ↓ MEP amplitude in the amputation group (vs baseline) specific to amputated arm–only left motor area (p = 0.025); no differences for right motor area (p = 0.15)	↓ corticomotoneuronal excitability
		Measured at baseline and after condition		No change in MEP amplitude in the control group (vs baseline) for left or right motor area (p = 0.89–0.93)	
				↑ ratings on amputation specific questions in amputation group (vs control); both groups experienced virtual body as their own (no difference for general body ownership questions)	

Within subject = repeated conditions delivered to the same group/participant; between subject = two or more groups receiving different conditions; DF, dorsiflexion; PF, plantarflexion; 1PP, first person perspective; TMS, Transcranial magnetic stimulation; RMT, Resting motor threshold; EMG, Electromyography; AMT, Active motor threshold; TA, Tibialis Anterior muscle; FDI, First dorsal interosseous muscle; ADM, Abductor digiti minimi muscle; FCR, Flexor Carpi Radialis Muscle; ECR, Extensor Carpi Radialis Muscle; ECU; Extensor Carpi Ulnaris Muscle/tendon; MEP, Motor evoked potential; ANOVA, Analysis of Variance; RM ANOVA, repeated measures ANOVA; d, Cohen’s d effect size estimate; VAS, Visual analogue scale; SP, Silent period; ↑, increase; ↓, decrease; abd, abduction; add, adduction; SICI, short-interval intracortical inhibition; ICF, intracortical facilitation; NMDA, N-methyl-D-aspartate ms, milliseconds; mV, millivolt; RHI, rubber hand illusion.

*Reported results only for illusion coil orientation RDB (side B of the coil facing up)

^Ʌ^ Illusion condition (but not for the primary purpose of the study)

^†^ Not a primary analysis of the study

### Kinaesthetic illusions

Five studies (7 experiments; total n = 87 participants) evaluated kinaesthetic illusions that conferred an illusory feeling of body movement via vision or via tendon vibration.

#### Effect of kinaesthetic illusions induced by vision

Three studies (five experiments) used non-immersive virtual reality where participants viewed a computer screen, spatially aligned to their own foot/hand position, showing a video of another person performing a foot/hand/finger movement from a first-person perspective [42, 50]. Such a set-up created an illusion that the participants’ own body part was moving.

Aoyama et al (2012) evaluated illusory ankle dorsiflexion (DF) and plantar flexion (PF), comparing both to a rest condition (unclear whether condition order was randomised) at 3 different TMS stimulus intensities (105%, 115%, and 125% of RMT). Increased corticomotoneuronal excitability was found during the DF illusion for TA (a muscle that dorsiflexes the ankle). MEP amplitudes (normalised to the maximal compound wave; termed Mmax) for TA were significantly higher in the DF illusion than in the rest condition for all intensities (see Table 3) and the PF illusion condition (for 105% and 115% RMT). In contrast, there was no effect on corticomotoneuronal excitability during the PF illusion: MEP amplitudes (normalised to the M-max) did not differ between conditions for the soleus muscle (plantar flexes the ankle). Of interest, the sensation of illusory ankle movement was perceived to be stronger during the DF illusion than during the PF illusion (p = 0.008; d = 1.10).

**Table 3:** MEP amplitudes (normalised to Mmax) and effect size for Aoyama et al (2012) [47].

Muscle tested & Condition	105% RMT			115% RMT			125% RMT		
	Mean	SD	Cohen’s d	Mean	SD	Cohen’s d	Mean	SD	Cohen’s d
**Tibialis Anterior**									
Rest	4.17	6.07	DF vs Rest: 0.88 DF vs PF: 1.05 PF vs Rest: 0.002	6.00	4.46	DF vs Rest: 0.82 DF vs PF: 0.49 PF vs Rest: 0.32	7.94	4.91	DF vs Rest: 0.65 DF vs PF: 0.52 PF vs Rest: 0.20
DF illusion	8.60	4.94		9.74	5.48		12.70	9.12	
PF illusion	4.18	4.46		7.42	5.21		8.89	5.49	
**Soleus**									
Rest	0.92	0.65	DF vs Rest: 0.075 DF vs PF: 0.46 PF vs Rest: 0.43	0.99	0.76	DF vs Rest: 0.14 DF vs PF: 0.53 PF vs Rest: 0.64	1.27	0.84	DF vs Rest: 0.17 DF vs PF: 0.071 PF vs Rest: 0.27
DF illusion	0.88	0.52		1.10	0.97		1.45	1.29	
PF illusion	1.41	1.40		1.66	1.30		1.53	1.21	

RMT, resting motor threshold; SD, standard deviation; DF, dorsiflexion; PF, plantarflexion. Cohen’s d for dependent samples. MEP, motor evoked potential, Mmax, maximal compound wave

In Kaneko et al. (2007) Experiment 1, illusory index finger abduction was compared with a resting condition and non-illusion control where participants could see their static hand (Group A) and with a resting condition and sham illusion control of non-biological movement of text (Group B) [42]. While the order of the illusion and non-illusion/sham control conditions was randomised, the resting condition was always assessed first (and was not re-assessed throughout the experiment). Further, in the analyses, the two groups were combined and analysed together using a one-way ANOVA (see Table 2), creating differences in group sizes and not accounting for the repeated nature of some comparisons. Experiment 2 compared identical illusory index finger abduction with additional finger movements on a video (index finger adduction, 5^th^ finger abduction/adduction). In both experiments, the participant’s index finger was positioned and held statically at 30° of abduction. In the illusion and control conditions, corticomotoneuronal excitability was measured when the index finger in the video moved to 30° abduction. In both experiments MEP amplitudes were significantly larger during the illusion conditions compared to all other conditions, indicating increased corticomotoneuronal excitability (See Table 4) [42]. Further, the increase in corticomotoneuronal excitability was specific to the muscle group of the finger undergoing the illusion in both experiments: excitability changed only for the FDI not the ADM. The MEP increase ratio (MEP amplitude during illusion condition/MEP amplitude at rest) differed significantly between FDI and ADM (t_1,19_ = 2.41, p = 0.026, d = 0.55).

**Table 4:** MEP amplitude (mean, standard deviation) and effect size, Kaneko et al (2007) [42].

Conditions	MEP amplitude:			
	FDI (muscle matched to illusion)		ADM (control muscle)	
	During	d	During	d
**Kaneko E1:**				
Illusion	6.45 ±4.35		4.91 ±4.14	
Rest	3.18 ±1.21	I vs R: 0.87	3.53 ±2.04	I vs R: 0.41
Non-illusion	3.42 ±1.56	I vs N: 0.82*	3.23 ±2.47	I vs N: 0.45*
Sham	2.26 ±1.20	I vs S: 1.15*	3.15 ±2.15	I vs S: 0.49*
**Kaneko E2:**				
Illusion (index-abd)	18.41 ±13.48		12.75 ±14.31	
Index–add	8.65 ±6.72	I vs I-add: 0.90	7.73 ±6.82	I vs I-add: 0.43
Little–abd	7.71 ±9.45	I vs L-abd: 0.98	8.20 ±9.20	I vs L-abd: 0.40
Little–add	5.73 ±6.62	I vs L-add: 1.17	5.67 ±6.52	I vs L-add: 0.60
Rest	6.52 ±5.77	I vs R: 1.08	6.76 ±6.13	I vs R: 0.51

MEP, motor evoked potential; FDI, first dorsal interosseous; ADM, Abductor digiti minimi; I, Illusion; R, Rest; S, Sham; I-add, Index-adduction; L-abd, Little finger abduction; L-add, Little finger adduction;

*Cohen’s d for independent samples; remainder are Cohen’s d for dependent samples.

Noijama et al (2015) (two experiments, n = 30) also used non-immersive virtual reality where participants watched a video of a ball rotation task (first-person perspective) to create an illusory sense of movement [50]. In experiment one, participants were randomised into one of two groups: 1) the rubber ball movement illusion; 2) viewing a static image of a hand holding two rubber balls (control). Measures were taken pre- and post-condition in each group. The second experiment replicated these comparisons, assessing excitability *during the illusion* while adding in an additional condition where the participant watched the rubber ball rotation task from a third-person perspective and also comparing to a rest condition (measured pre- and post-experiment). Consistent with the study above [42], there was an increase in MEP amplitude specific to the illusion (first-person perspective) conditions in both experiments and these effects were large (see Table 5). In Experiment 1, the illusion and static control condition differed, while in Experiment 3, the illusion condition differed only from outcomes taken while at rest (and no other conditions–static or 3^rd^-person perspective–differed from rest). Further, paired pulse stimulation was used to evaluate the effect of the illusion on short-interval intracortical inhibition (SICI; strength of GABA_B_-mediated intracortical inhibition) and intracortical facilitation (ICF; strength of glutamatergic N-methyl-D-aspartate [NMDA] receptor mediated facilitation) within the motor cortex in both experiments [50]. There was no effect of the illusion on SICI; however, ICF increased post-intervention in the illusion condition compared with the control condition (static hand) in Experiment 1 and with the rest condition in Experiment 2.

**Table 5:** MEP amplitude, SICI, and ICF (mean +/- standard error), effect sizes, and analysis results for Noijma et al (2015) [50].

Conditions	MEP			SICI			ICF		
	Pre/During†	Post	d	Pre	Post	d	Pre	Post	d
**E1:**									
Illusion (n = 10)	687.4 ±133.0	938.2 ±205.5	0.48	0.504 ±0.054	0.520 ±0.079	0.12	1.202 ±0.071	1.549 ±0.119	1.15
Static (n = 9)	678.0 ±122.5	680.2 ±128.6	0.007	0.492 ±0.062	0.417 ±0.055	0.47	1.353 ±0.111	1.293 ±0.087	0.22
I vs S (Post)	---	---	0.48*	---	---	0.49*	---	---	0.79*
Group x Time	---	---	2.11*	---	---	N/A	---	---	1.25
**E2 (n = 20):**									
Illusion	847.4 ±160.9	N/A		0.550 ±0.068	N/A		1.368 ±0.074	N/A	
Rest	506.6 ±67.9	N/A	I vs R: 0.82	0.460 ±0.069	N/A	I vs R: 0.47	1.192 ±0.058	N/A	I vs R: 0.92
Static	556.1 ±93.4	N/A	I vs S: 0.72	0.548 ±0.071	N/A	I vs S: 0.01	1.210 ±0.056	N/A	I vs S: 0.83
3^rd^ person	792.2 ±164.5	N/A	I vs 3^rd^: 0.12	0.487 ±0.064	N/A	I vs 3^rd^: 0.34	1.208 ±0.052	N/A	I vs 3^rd^: 0.85

E1 = Experiment 1; E2 = Experiment 2; d = Cohen’s d; RM = repeated measures; ANOVA = analysis of variance; I = illusion; S = static; R = rest; 3^rd^ = 3^rd^ person Group x Time, Group x Time interaction from 2 way repeated measures ANOVA. MEP, motor evoked potential; SICI, short intra-cortical inhibition; ICF, intracortical facilitation

^**†**^ Measures were taken pre-condition in E1 and during the test condition in E2.

* Cohen’s d for independent samples; the remaining are Cohen’s d for dependent samples.

#### Interim discussion

Concerns with randomisation and analysis in Kaneko et al (2007) question whether increases in corticomotoneuronal excitability seen with the kinaesthetic illusion vs baseline resting levels were due to the illusion or due to a confounding factor, such as time. However, that the illusion and non-illusion conditions (order randomised) differed in the study by Kaneko et al (2007) and that similar effects on corticomotoneuronal excitability were shown by Noijma et al (2015) (order randomised) increases confidence in these results. Aoyama et al’s (2012) finding suggest that such effects of illusory movement on corticomotoneuronal excitability are not limited to the upper limb, but also extend to the lower limb. That the DF illusion was more intensely experienced than the PF illusion and that only the DF illusion altered corticomotoneuronal excitability suggests a link between illusion strength and influence on the motor cortex. However, concerns with randomisation in Aoyama et al (2012) suggest that this result requires replication.

Noijma et al (2015) showed that visual kinaesthetic illusions may have differential effects on inhibition and facilitation within the motor cortex. While there was no significant effect of illusory movement on SICI, the effect sizes for some comparisons were moderate in size (d = 0.34–0.49), which raises the possibility that it may be underpowered. However, that effects of illusory movement on corticomotoneuronal excitability are specific to the muscle consistent with the movement illusion is supported by both Kaneko et al (2007) and Aoyama et al (2012). Clearly further work is needed to elucidate such findings, namely with studies using assessor blinding, and robust (and preferably pre-registered) randomisation procedures and statistical analyses. Despite small sample sizes, the effect sizes seen were generally moderate to large.

#### Effect of kinaesthetic illusions induced by tendon vibration

Two studies used high frequency tendon vibration to induce an illusory sense of limb movement [51, 52]. Data were unavailable for effect size calculation.

Mancheva et al (2017) provided Flexor Carpi Radialis (FCR) tendon vibration to induce an illusory feeling of wrist extension [51]. Corticomotoneuronal excitability was assessed during low amplitude vibration, and high amplitude vibration during 3 conditions: eyes closed, eyes open but no vision of the wrist, and eyes open with vision of the wrist [51]. High amplitude vibration with the eyes closed or eyes open (but no vision of the wrist) were the illusion conditions. Additionally, excitability was assessed during a no vibration condition (eyes open) at baseline. Corticomotoneuronal excitability was assessed before and during the illusion as well as after the illusion to also evaluate the post-vibration inverse illusion that occurs (i.e., a feeling of illusory flexion of the wrist as the extension illusion dissipates). High amplitude vibration of the FCR tendon resulted in increased Extensor Carpi Radialis (ECR) MEP area only during the eyes open (no vision of wrist) illusion condition (i.e., consistent with an illusory feeling of wrist extension), and while the FCR MEP area increased from baseline (tendon being vibrated), it did so in all conditions (vs no vibration controls). Interestingly, while the second illusion condition (high amplitude vibration and eyes closed) did induce an illusion of wrist extension, there was no increase in ECR MEP area for this area. Regardless, such results suggest a specific effect on corticomotoneuronal excitability only for the muscle consistent with the illusory movement direction and consistent with the illusion experience given that low amplitude vibration (no illusion) did not affect ECR MEP area (vs no vibration controls). An increase in FCR MEP area with low amplitude vibration (versus rest, p<0.05) was consistent with application of vibration, but FCR MEP area did not differ between test conditions. Additionally, FCR MEP area did not differ between low and high amplitude vibration of the FCR tendon (p-values not reported). During the post-vibration inverse illusion (i.e., illusory feeling of wrist flexion) there were no effects on ECR MEP area, and inconsistent effects on the FCR MEP area (muscle consistent with illusory movement). The FCR MEP area (consistent with inverse illusion) was significantly decreased during eyes open (no vision of wrist) illusion condition, but was significantly increased during both closed eyes (illusion) condition and the wrist watching (control) condition.

Naito et al (2002) evaluated the effect of an illusory sense of wrist flexion using tendon vibration of the wrist Extensor Carpi Ulnaris (ECU) tendon, but with both hands placed together, palm on dorsum (i.e., evaluating an illusion transfer) [52]. Corticomotoneuronal excitability of the non-vibrated hand was assessed when the hands were in contact and when the hands were separate and used three levels of vibration (no vibration; high amplitude vibration; low amplitude vibration) resulting in six conditions. High amplitude vibration with hands in contact was the illusion condition. The MEP amplitude from the wrist flexors of the non-vibrated hand during the illusion condition was significantly larger (and increased from baseline) than all other 5 conditions (i.e., consistent with an illusory feeling of wrist flexion). It was also found that MEP amplitude from the wrist extensors of the non-vibrated hand was significantly reduced during the illusion condition (i.e., consistent with reduced activity that would be needed during actual wrist flexion movement). Both MEP amplitude changes occurred only when the subjects reported experiencing the onset of illusory flexion of the non-vibrated wrist.

#### Interim discussion

Taken together, the above findings suggest that illusory limb movement (induced by tendon vibration) has muscle and condition-specific effects–stimulation of the motor area of the antagonist muscle to the illusory movement direction was found to elicit increased corticomotoneuronal excitability in illusion versus non-illusion control conditions. In contrast, condition-specific effects do not exist in the muscle receiving the tendon vibration. That is, while tendon vibration elicits increased corticomotoneuronal excitability for its respective muscle’s motor area, it does so regardless of the test condition. Further studies are clearly needed, particularly given that the present studies were at high risk of bias for statistical methods, with many of the primary analysis findings not reported (e.g., only post-hoc findings reported [51]), and small sample sizes.

It is interesting that in Mancheva et al (2017) only one of the illusion conditions–high amplitude vibration with eyes open but no vision of the hand–elicited increased corticomotoneuronal excitability in the motor area of the muscle (ECR) consistent with the direction of illusory movement. The other illusion condition–high amplitude vibration with eyes closed–did not elicit increased corticomotoneuronal excitability, despite inducing a feeling of illusory movement. This result may be explained by the influence of vision–during low amplitude vibration of the FCR tendon (no illusion), ECR MEP area was reduced (versus rest) only when the eyes were closed; this suppression did not occur in either of the eyes open conditions. It is possible that such suppression with eyes closed counteracted any facilitatory effects of the visual illusion. These findings suggest a role for both vision itself and illusory movement influencing excitability of the motor cortex and its descending projections to the muscle.

### Rubber hand illusions

Five studies (6 experiments; total n = 87 participants) evaluated the effect of a rubber hand illusion (RHI) on corticomotoneuronal excitability, with one study also evaluating the strength of GABA_B_-mediated intracortical inhibition within the motor cortex (i.e., silent period duration).

#### Effect of visuotactile RHI

Three studies evaluated the effect of a traditional RHI where synchronous visuotactile input is provided (i.e., both the real hand and the rubber hand are synchronously stroked) [41, 48, 49]. The results were conflicting when the embodied hand was static, whereas more consistent results were observed when evaluating the effect of embodying another’s hand and then observing that hand moving.

della Gatta et al (2016) found a significant decrease in raw MEP amplitude (mean ± SD) in the RHI condition (i.e. synchronous stroking: (0.53±0.33mv) compared with the asynchronous control condition (0.92±0.64mv; p = 0.00092, d = 0.85) and the baseline condition (0.95±0.55mv; p = 0.0017, d = 0.74) [41] in participants who embodied the rubber hand (i.e., disembodied their real hand). The effect was also specific to the hand undergoing the RHI–there were no significant differences in raw MEP amplitude between conditions (synchronous, asynchronous, baseline) for the real, unstimulated hand that was not undergoing the RHI (p = 0.42; d = 0.22–0.26).

Schutz-Bosbach et al (2006) applied the principles of the traditional RHI to a paradigm that used the experimenter’s hand in place of a rubber hand (i.e., embodiment of an experimenter’s static hand). This study also evaluated the effect of movement observation (the experimenter moving their hand) when the participants embodied the experimenter’s hand (synchronous stroking: ‘self action’) and when they did not (asynchronous stroking: ‘other person action’). When the participants embodied the experimenter’s hand and observed the experimenter’s hand moving (synchronous ‘self action’), the FDI MEP amplitude normalised to baseline (mean +/- SEM) was suppressed (1.46±0.23) relative to observation of a static hand (synchronous: 1.56±0.28, d = 0.11, p-value not reported). When participants did not embody the experimenter’s hand (asynchronous ‘other action’), and observed it moving, the FDI MEP amplitude normalised to baseline increased (1.44 ±0.10) relative to observation of a static hand (asynchronous: 1.37 ±0.12; d = 0.16, p-value not reported) [48].

While not directly calculated in the paper [48], the FDI MEP amplitudes normalised to baseline were unlikely to have differed between self-action (embodying the hand, watching it move) and other-action (not embodying the hand, watching it move) given small effects seen (d = 0.028; see Table 6); rather an interaction was seen between ownership and viewed movement (p = 0.023). Such findings were specific to the FDI (muscle consistent with viewed movement): the ADM muscle did not show a significant interaction between conditions of ownership and viewed movement (p = 0.68), although main effects of ownership appear present (results not reported; see Table 6). Last, while not a primary comparison of the paper, it appears that there was either no difference or an increase in the FDI MEP amplitude normalised to baseline between the synchronous static hand condition (i.e., traditional RHI) and asynchronous static hand condition (p-value not available; d = 0.13). Such findings are in contrast to the previous study above [41].

**Table 6:** MEP amplitudes and effect sizes measures for Schutz-Bosbach et al. (2006) [48].

Muscle tested/Condition		MEP amplitude		Cohen’s d		
		Mean	SE	Primary illusion: Self-action	Secondary illusion: Traditional RHI (Self-static)	Other-action:
**FDI**						
Synch/Self	Static hand	1.56	0.28	Self-action vs: Self-static: 0.11 Other-static: 0.13 Other-action: 0.028	Self-static vs: Other-static: 0.15	Other-action vs: Other-static: 0.16 Self-static: 0.15
	View action	1.46	0.23			
Asynch/Other	View action	1.44	0.11			
	Static hand	1.37	0.12			
**ADM**						
Synch/Self	Static hand	1.30	0.19	Self-action vs: Self-static: 0.034 Other-static: 0.36 Other-action: 0.41	Self-static vs: Other-static: 0.34	Other-action vs: Other-static: 0.027 Self-static: 0.36
	View action	1.28	0.16			
Asynch/Other	View action	1.10	0.11			
	Static hand	1.11	0.11			

Synch, synchronous; Asynch, asynchronous; MEP, motor evoked potential; SE, standard error. Cohen’s d for dependent samples calculated;

Schutz-Bosbach et al (2009) evaluated the effect of the RHI on the cortical silent period (i.e., strength of GABA_B_-mediated intracortical inhibition within the motor cortex) and MEP amplitude while participants maintained isometric tonic contraction of the FDI [49]. Similarly, they used an experimenter’s hand and evaluated two factors: embodiment (i.e., synchronous vs asynchronous stroking) and action observation (experimenter’s moving vs static hand). During synchronous stroking, the mean cortical silent period duration (mean +/-SEM) was significantly longer when participants viewed hand actions (162±11 ms) versus when they observed a static hand (159±11 ms; d = 0.39), indicating stronger GABA_B_-mediated intracortical inhibition within the motor cortex. These effects were reversed in the asynchronous stroking condition: when subjects viewed hand actions (not attributed to themselves), mean cortical silent period duration was significantly shorter (153±11ms) than when subjects observed a static hand (157±11 ms; d = 0.66). Lastly, silent period duration in the synchronous stroking hand action condition was significantly longer (162±11ms) than the asynchronous stroking hand action condition (153±11 ms; d = 1.12). There were no differences in cortical silent period between the synchronous (traditional RHI) and asynchronous static hand conditions (p = 0.09, d = 0.14), although the synchronous condition (RHI) differed from baseline (p = 0.042), while the asynchronous condition did not (p = 0.12). Because the silent period was the primary outcome measure for this study, the experimental set-up resulted in 5.9% of the MEPs not being measurable (because the top and/or bottom part of the MEP was missing). Therefore, the lack of difference seen in FDI MEP amplitudes between conditions is taken with caution.

#### Interim discussion

That conflicting results were seen for corticomotoneuronal excitability during the static visuotactile RHI merit discussion. First, such differences may reflect differences in RHI paradigm (rubber hand vs experimenter’s hand, what conditions are used to measure baseline excitability), but are most likely due to differences in TMS protocol (e.g., TMS stimulation during rest [41] versus tonic submaximal contraction [48, 49]) and stimulation intensity (e.g., an intensity of 110% of RMT [41] vs 130% of AMT [49] vs 105% of RMT [48]). Because of differences in stimulus intensity used it is likely that current density, penetration and volume of stimulated cortex varies between these studies [56]. These differences impact physiological mechanisms that underpin MEP amplitude, such as the number of motor neurons recruited in the spinal cord, the number that discharge more than once to the stimulus, and the synchronisation of the discharged motor neurons [56]. The studies’ participants were largely similar in age and gender ratio, suggesting that participant features were unlikely to have contributed to the differences in results. Second, the studies were largely similar with respect to risk of bias in that all studies used randomisation and recruited naïve participants (although given that blinding was not assessed, we cannot be certain participants were truly blinded to condition). However, only della Gatta et al 2006 performed an *a priori* sample size calculation and thus was appropriately powered for any comparisons, which increases the relative weighting given to their findings that disowning your own hand (via RHI) decreases corticomotoneuronal excitability [41].

The results for embodying another person’s hand (via visuotactile RHI) and seeing that embodied hand move (while your hand is still static) were largely consistent between two studies [48, 49]. Viewing others’ actions (i.e., not embodying the hand) had a facilitatory effect on the motor system (increased corticomotoneuronal excitability and reduced inhibitory activity in M1), whereas viewing actions linked to the self (i.e., embodying the hand), resulted in relative decreases in cortical excitability and increase inhibitory activity in M1. Given that full results were not reported for such effects on a muscle not involved in the illusion (ADM) [48], it cannot be concluded that the effects on corticomotoneuronal excitability of embodying a hand and viewing movement are specific to the muscle consistent with the viewed movement.

#### Effect of visuomotor RHI

Two studies evaluated a motor version of the RHI that involved participants moving their own hands. Weiss et al (2014) (n = 12), evaluated excitability when active movement of the participant’s finger was paired with either synchronous visual input (i.e., accurate) or asynchronous visual input (i.e., delayed movement) [54]. The visual input was presented in a spatial location separate to the real hand, which induces a manipulation of ownership/agency (sense of control over the viewed movement). Perceptual ratings confirmed that these conditions evoked a feeling of agency for the synchronous condition but not for the asynchronous condition. There was a significant influence of level of delay on corticomotoneuronal excitability: in conditions of temporal delay (i.e., asynchronous) there was a significant increase in the MEP amplitude normalised to baseline as compared with the synchronous condition that evoked a sense of agency (See Table 7). That is, the illusion of agency resulted in a *relative* decrease in corticomotoneuronal excitability vs conditions in which agency wasn’t present. However, it is noted that the illusion of agency condition had an absolute increase in excitability versus baseline levels (p-value not reported). Further, there was a positive correlation between delay and MEP amplitude normalised to baseline (r = 0.40): the greater the temporal delay, the greater the normalised MEP amplitude (i.e., increased corticomotoneuronal excitability). Last, delay had no influence on corticomotoneuronal excitability of a control muscle (ADM) that was unrelated to the executed/observed movement (p = 0.69).

**Table 7:** Mean MEP amplitudes, statistical results, and effect sizes for Weiss et al (2014) [54].

Condition	MEP amplitude normalised to baseline (mV)		Statistical results	Cohen’s d for illusion
	Mean	SEM		
0 ms (illusion)	1.89	0.21	0 vs 100 ms: t_28_ = 2.53, p = 0.017 100 vs 200 ms: t_28_ = 0.18, p = 0.86 200 vs 300 ms: t_28_ = 2.13, p = 0.42	0 vs 100 ms: 0.092 0 vs 200 ms: 0.087 0 vs 300 ms: 0.17
100 ms	1.99	0.24		
200 ms	1.98	0.22		
300 ms	2.07	0.24		
**Ambiguous stimuli (delay of 100 ms and 200 ms)**				
Judged as corresponding	1.99	0.31	2 (delay) x 2 (correspondence) RM ANOVA Main effect of correspondence: F_1,11_ = 5.85, p = 0.34	0.21
Judged as non-corresponding	2.19	0.32		

MEP, motor evoked potential mV, millivolts; ms, milliseconds; RM ANOVA, repeated measures analysis of variance, Cohen’s d for dependent samples calculated.

Additionally, Weiss et al (2014) considered the influence of explicit recognition of delay in ambiguous situations (e.g., a small visual delay for which participants sometimes recognised or failed to recognise non-correspondence) [54]. When correspondence judgements, level of delay, and corticomotoneuronal excitability were considered together, significant correlations were found (visual delay and correspondence, r = -0.87; correspondence and excitability, r = 0.41; see above for delay and excitability). Analyses showed that when the executed movements were judged as not corresponding to one’s own executed movement (using 100 ms and 200 ms delay–most ambiguous) there was a significant increase in the MEP amplitude normalised to baseline than when the executed movements were judged as corresponding to one’s own executed movement (p = 0.034; Table 7).

In Karabanov et al (2017), a customised experimental set-up was used where the participant’s left hand was positioned underneath (but not touching) two gloved rubber hands [53]. One of the gloved hands was spatially aligned with the anatomical position of the real hand. The other gloved hand was positioned facing the participant as if it were someone else’s hand (anatomically implausible). Participants were asked to move their left index finger. Three conditions were tested: agency and ownership (anatomically aligned rubber hand moved in time with real finger movement); agency no ownership (the anatomically implausible rubber hand moved with the real finger), no agency and no ownership (neither of the hands moved). There was no difference in MEP amplitudes (p = 0.11), and thus corticomotoneuronal excitability, between conditions [53]. Data were unavailable for effect size calculation.

#### Interim discussion

While the two study results find conflicting effects of altering agency, differences in study size and TMS testing paradigm may underpin the differences seen here. It is plausible that Karabanov et al’s finding of no effect of agency or ownership on corticomotoneuronal excitability [53] may be a feature of low sample size such that it is underpowered to detect a difference between conditions (n = 7). Moreover, the TMS stimulus intensity in Karabanov et al was provided at 100% of RMT [53] (versus 110% of RMT in Weiss et al [54]), which may increase the variability of MEPs [57] and reduce the ability to detect between condition differences. Given that Weiss et al recruited a larger number of participants (n = 29), had a lower overall risk of bias, and used TMS stimulation intensities (110% RMT) that produce less variable MEPs [57], more weight is placed in these results.

### Missing limb illusions

One study created an illusion of a missing or amputated arm [55]. It was theorised that congruent visuotactile information that provides compelling information that a body part is missing (and thus cannot be used in movement) would result in a perceptually mediated reduction in sensorimotor representation in the brain. Using a virtual reality set-up, one group received the illusion of an amputated arm by seeing an avatar with the right arm missing (and seeing the location where the arm should be on the table being touched and hearing taps on the underlying table). The second group saw an intact avatar and felt touch on their own arm when the virtual avatar’s arm was touched (control group). MEP amplitudes were measured before and after each condition for both the right (illusion) and left (control) hand. No effects of the illusion were found on FDI MEP amplitudes (p>0.28), but the interaction between group, hand, and condition approached significance for ECU MEP amplitudes (p = 0.053, d = 0.73). Exploratory post-hoc tests showed a significant decrease in ECU MEP amplitude measured contralateral to the illusory amputated arm (versus baseline, p = 0.025; d = 0.48). No differences in MEP amplitudes were found in the control group (full body avatar) before and after the virtual reality session for either hemisphere (left: p = 0.189, d = 0.029; right: p = 0.93, d = 0.02) nor in the MEP amplitude measured ipsilateral to the illusory amputated arm in the illusion group (p = 0.15; d = 0.30). The lack of *a priori* sample size estimates, combined with moderate-to-large, but non-significant statistical results, raise the possibility that this study was underpowered.

## General discussion

This review found a limited number of studies (n = 11) evaluating the effect of bodily illusions on corticomotoneuronal excitability. All studies evaluated corticomotoneuronal excitability, while two experiments also evaluated intracortical inhibition and one experiment evaluated intracortical facilitation in the motor cortex. All included studies had a high risk of bias, with low to moderate quality of TMS reporting. Despite these limitations, there was evidence that perception of illusory movement of your own limb (i.e., kinaesthetic illusions of movement) increased corticomotoneuronal excitability, specifically in the illusion condition and for the muscle group corresponding to the direction of the movement illusion. While tendon vibration itself increased corticomotoneuronal excitability for the muscle being vibrated, this occurred regardless of the condition.

Interestingly, the opposite effect of illusory movement was seen when the limb was disowned. In visuotactile RHI paradigms, embodying a hand and then seeing it move (illusory self-action) has an inhibitory effect: it reduces corticomotoneuronal excitability and increases inhibition (i.e., silent period duration: GABA_B_-mediated intracortical inhibition within the motor cortex). In contrast, not embodying a hand and then seeing it move (other-action) has a facilitatory effect: it increases corticomotoneuronal excitability and decreases inhibition (i.e., silent period duration). Such results are supported by visuomotor RHI findings showing increased corticomotoneuronal excitability as a function of increasingly delayed observed movement (becoming more ‘other-action’ than ‘self-action’). Conflicting results were found for the traditional visuotactile static RHI although differences seen likely reflect variations in methodology. Last, a single study found that an illusion of an amputated limb reduces corticomotoneuronal excitability. Together these findings have important theoretical implications.

### Illusory limb movement via kinaesthetic illusions

This review found evidence that, regardless of the mode of induction (vision or tendon vibration), kinaesthetic illusions that induce the feeling of limb movement increase corticomotoneuronal excitability. That such changes are due to the illusion (and not merely sensory input) are supported by both past work and the present findings. First, the changes in corticomotoneuronal excitability seen here were specific to the muscle whose action was consistent with the direction of illusorily induced movement (‘illusion agonist muscle’), regardless of mode of induction [42, 47, 50–52]. Second, changes in excitability for the illusion agonist muscle were seen only during the illusion condition [42, 47, 50–52]. Third, sensory input alone (vision or tendon vibration) did not influence corticomotoneuronal excitability for the illusion agonist muscle. For example, in visual kinaesthetic illusions, seeing an identical hand movement, but not in a manner inducing an illusion, did not change MEP amplitudes from resting measures for the illusion agonist muscle [50]. For tendon vibration, when the illusion was not induced, there were no changes in corticomotoneuronal excitability for the illusion agonist muscle (and this was supported by the lack of condition-specific effects in the muscle receiving vibration) [51]. Importantly, increases in corticomotoneuronal excitability for the illusion agonist muscle were shown to occur even in the absence of tendon vibration to the hand experiencing the illusion (i.e., during illusion transfer) [52].

That corticomotoneuronal excitability changes reflect the illusion induced and not merely the sensory input applied are supported by previous neuroimaging work. For example, distinct brain activation patterns (measured using fMRI) are seen when one is experiencing a kinaesthetic illusion compared with merely watching that same movement [58]. In addition, during tendon vibration-induced kinaesthetic illusions, increased activation of the contralateral somatosensory and primary motor cortex (M1) occurs [59] but M1 activation, (measured using positron-emission tomography), is higher during kinaesthetic illusion than during tendon vibration alone [60].

The mechanisms underlying kinaesthetic illusions induced via tendon vibration or via vision are clearly different, in so far as that they involve unique initiating neural inputs. Therefore, that both types of kinaesthetic illusions have similar effects on excitability suggests that the unique sensory representations of limb movement–i.e., a proprioceptive representation and a visual representation of movement–can each similarly influence excitability of the motor cortex and its projection to the muscle. These findings suggest involvement of higher-order multisensory areas that integrate input from vision and proprioception, such as the premotor cortex [61, 62] and/or the posterior parietal cortex [63, 64], in influencing corticomotoneuronal excitability. Such modulation by multisensory areas is possible: rTMS to alter excitability of the premotor cortex has been shown to induce changes in corticomotoneuronal excitability [65]. Moreover, illusion- and muscle-specific corticomotoneuronal excitability changes during tendon vibration occur even when the brain receives no direct muscle spindle afferent input signalling the limb’s movement (i.e., as a result of an illusion transfer to the non-vibrated hand when the hands are in contact [52]). Such findings support the idea of a higher order influence, rather than merely a low-level sensory influence, on corticomotoneuronal excitability, that is likely also underpinned by communication between sensorimotor networks of the limbs (i.e., reconciling the input that one wrist is bending with the input that both hand are in contact). Together, these findings support the presence of complex interactions between perceived movement and corticomotoneuronal excitability.

The present findings also raise the possibility that kinaesthetic illusions may have specific effects on numerous mechanisms underlying motor cortex function. Increases in corticomotoneuronal excitability during illusory movement (via tendon vibration) were seen concurrent with increases in facilitation (ICF), but in the absence of changes in inhibition (SICI) [50]. Past work has shown that SICI reflects excitability in GABA-A mediated interneurons in the motor cortex [66]. Thus, an increased MEP amplitude with unchanged SICI may suggest that decreased GABA-A mediated intracortical inhibition (i.e. decreased excitability of one type of interneuron in the motor cortex) is unlikely to contribute to the net increase in excitability of the pathway. The mechanisms that underlie ICF are not well understood, so it is unclear whether this may contribute to the net increase in motor pathway excitability. More work is clearly warranted, given this was explored by only one study.

### Illusions of body ownership and agency

Taken together, the present review suggests a unique effect of disowning your own hand on corticomotoneuronal excitability. During real movement, corticomotoneuronal excitability is increased. While visual kinaesthetic illusions that induced a feeling of self-limb movement *increased* corticomotoneuronal excitability, consistent with real movement, disowning your real hand and then seeing the embodied hand move (illusory self-action) resulted in a relative *decrease* in corticomotoneuronal excitability (reduced MEP amplitudes and increased GABA_B_-mediated intracortical inhibition) compared with asynchronous stroking (do not disown your real hand–viewing other-action). The MEP amplitude and silent period findings are therefore consistent with a picture of facilitation of the motor system when observing others’ actions (asynchronous visuotactile) and a relative reduction in corticomotoneuronal excitability when the same observed actions are illusorily attributed to their own body (synchronous visuotactile). The best available evidence (only *a priori* powered study [41]) suggests that disowning your own hand reduces corticomotoneuronal excitability (decreased MEP amplitudes). That similar reductions in corticomotoneuronal excitability occur when you ‘lose’ your own hand (via missing arm illusion) [55], provides further confidence in this result. Such findings raise the possibility that the absence of a facilitatory effect on the motor system after synchronous stimulation and viewed movement (‘self-action’) is due to a reduction in corticomotoneuronal excitability induced by embodiment of the rubber hand.

This review found evidence from both visuotactile and visuomotor RHI paradigms that when movement is perceived to *not* be your own, corticomotoneuronal excitability increases. For example, when agency over a moving finger was present, MEP amplitudes were increased from baseline levels, but when agency was broken via visual delay of finger movement, MEP amplitudes were significantly higher than during the agency condition [54]. Such effects are similar to those seen in conditions in which ownership is absent. That is, *not* disowning your real hand and then seeing the rubber hand move (other person action) also results in increased corticomotoneuronal excitability [48, 49]. However, while these effects of ownership likely reflect similar mechanisms that underlie action observation of another’s movement (which also increases corticomotoneuronal excitability [19, 33]), the agency illusion evaluated here may reflect mechanisms of incongruence detection. Previous work using a modified mirror feedback set-up to induce vision and movement incongruence found that the slower the observed movement (vs real movement), the greater the increase in MEP amplitude [67]. Similarly, when agency was manipulated by temporally delaying seen movement from real movement, corticomotoneuronal excitability positively correlated with the amount of delay [54]. Together, such findings suggest that corticomotoneuronal excitability may reflect a measure of agency (i.e., excitability changes may indicate one’s perceived control over a movement). That agency effects were specific to the muscle whose action is consistent with the direction of illusory movement (i.e., differences for FDI but not ADM [54]) supports this contention.

Of interest are preliminary findings that explicit awareness of agency as induced by illusions might influence the excitability of the motor cortex and its projections. Specifically, in ambiguous situations of small visual delay between seen and actual movement, corticomotoneuronal excitability (via MEP amplitudes) was relatively reduced for trials in which visual input was judged as corresponding (agency) versus non-corresponding (no agency) to actual movement [54]. This raises two main possibilities: first, that higher-order explicit representations of agency are a ‘read-out’ of lower-order sensorimotor input, meaning that levels of corticomotoneuronal excitability determine explicit agency. Second, it may be that higher-order explicit representations of agency are informed by numerous different agency cues (e.g., sensorimotor input, but also by other features such as interoceptive state [68]) as supported by Bayesian cue integration theory perspective [69]. Violation of agency is thought to occur when the prediction error between intended and perceived movement is sufficiently large [30]. A Bayesian perspective supports that prior knowledge/experience (top-down features) can be integrated by the model (i.e., our expectations of the results become priors for the level below, suggesting top-down influences can occur) [69]. Attention promotes neural encoding of prediction error [70], and is often drawn by unexpected outcomes (e.g., violated agency)–such findings would predict increased corticomotoneuronal excitability, given past work showing that focussed attention enhances increases in corticomotoneuronal excitability during movement observation [71].

### Strength and limitations

This review used gold-standard methodology for systematic reviews [43, 45] with a planned protocol decided *a priori*. The search strategy used was sensitive and all processes of the review (screening, eligibility, bias assessment, data extraction) were completed by two independent authors. Dual assessment by independent reviewers and hand searching of the reference lists of all full text studies, reduces the risk of missing potentially eligible studies. Additionally, the majority of studies adequately described the illusion, allowing for future replication testing.

The conclusions of this review are limited in that all included studies had a high risk of bias and none were of high-level evidence (i.e. randomised controlled trials). Sample sizes of included studies were typically small (mean, SD: 16 ±9), which increases the risk of spurious findings. Furthermore, the wide variety of illusions, comparison conditions, and TMS methodologies used prevented meta-analyses.

Given that the present review focussed on the effect of illusions on corticomotoneuronal excitability in healthy volunteers, the current findings clearly have very limited generalisability to clinical populations for therapeutic use. However, that use of non-invasive brain stimulation paradigms to alter corticomotoneuronal excitability have shown evidence of positive effect for movement and pain [9–11] suggests that further investigations of illusory effects on corticomotoneuronal excitability in clinical populations are warranted.

### Future research

Replication studies that use high-quality methodology to evaluate the effect of bodily illusions on corticomotoneuronal excitability are needed. Specifically, the most common forms of bias found by this review were a lack of blinding of participants/researchers and use of small sample sizes. Addressing these methodological issues will reduce bias, thus allowing stronger conclusions. Given the consistent effect of kinaesthetic illusions (induced by vision or tendon vibration) on increasing corticomotoneuronal excitability, future studies could extend use of these illusions to clinical conditions in which altered corticomotoneuronal excitability is present to determine if similar effects to those in healthy volunteers are seen. Additionally, it is known that individual differences exist in the degree to which illusions are experienced–for example, some people do not experience the RHI [72]. Specifically recruiting such individuals would be helpful to further explore the effects of illusions on corticomotoneuronal excitability. If people who do not experience limb disownership also do not have changes in corticomotoneuronal excitability (despite synchronous tactile and visual input), this would even more strongly implicate a higher-order influence, induced by the illusion, in driving excitability changes. Finally, exploring whether these illusions could be used as an adjunct to current therapy that aims to increase movement, particularly in a population in which actual movement is difficult or impossible (e.g., stroke rehabilitation), would be of interest.

## Conclusion

This systematic review found very low-level evidence for the effect of bodily illusions on corticomotoneuronal excitability, given high risk of bias and low overall numbers of eligible studies. Despite this, kinaesthetic illusions consistently increased corticomotoneuronal excitability specific to the muscle group undergoing the movement illusion. Conflicting effects were found for a static RHI. However, an illusory feeling of self-movement, via embodying a fake hand and watching it move, consistently decreased corticomotoneuronal excitability and increased GABA_B_-mediated intracortical inhibition within the motor cortex relative to observing other-action (i.e., not embodying the hand and then observing the hand move), which increased excitability and reduced inhibition. That RHI effects on corticomotoneuronal excitability differ than those resulting from kinaesthetic illusions of movement suggests that embodiment has unique effects on corticomotoneuronal excitability. Lastly, a reduction in corticomotoneuronal excitability was found during an illusion of a missing limb. The findings are preliminary and high-quality research is needed to better understand the mechanisms underlying perceptually-induced changes of the motor system.

## Supporting information

S1 Checklist PRISMA 2009 checklist. (DOC)

S1 File Medline search strategy. (DOCX)

S2 File Risk of bias assessment form. (DOCX)
