# Supplementary material for: What is the effect of bodily illusions on corticomotoneuronal excitability? A systematic review
Source: PLoS One. 2019 Aug 15;14(8):e0219754. doi: 10.1371/journal.pone.0219754 (PMC6695177; doi:10.1371/journal.pone.0219754)
Supplement: S1 File — (DOCX) [file pone.0219754.s002.docx]

**Supplementary Appendix 1**

**Medline search strategy**

(Perceptual Distortion/ or perceptual* distort* or perceptual illusion* or Illusions/ or illusion* or illusory or virtual reality)

AND

(Motor Evoked Potentials or MEP or inhibition or intracortical inhibition or short-interval intracortical inhibition or SICI or intracortical facilitation or Cortico-spinal excitability or motor cortical excitability or cortical excitability or corticospinal excitability)
