# Supplementary material for: What is the effect of bodily illusions on corticomotoneuronal excitability? A systematic review
Source: PLoS One. 2019 Aug 15;14(8):e0219754. doi: 10.1371/journal.pone.0219754 (PMC6695177; doi:10.1371/journal.pone.0219754)
Supplement: S2 File — (DOCX) [file pone.0219754.s003.docx]

**Risk of Bias Checklist**

*Reviewer:*

*Article:*

| **Type of Bias** | **Explanation** | **Risk of Bias** |
| --- | --- | --- |
| ***Selection Bias*** | Was the method of participant selection appropriate? (i.e. convenience sampling vs random sampling) | High risk of bias? Y/N  Why? (convenience)  Unclear risk of bias? Y/N  Why? (don’t say)  Low risk of bias? Y/N  Why? (random) |
|  | Other comments: | |
| ***Bias Related to study design*** | If within-subject study design:  Were study conditions performed in a randomised, counterbalanced order? | High risk of bias? Y/N  Why? (if it is not clear from methodology that it was randomised, and/or if it was randomised but not counterbalanced)  Unclear risk of bias? Y/N  Why? (don’t state it)  Low risk of bias? Y/N  Why? (state it) |
|  | If between-subject study design  Were participants randomly allocated into groups? | High risk of bias? Y/N  Why? (if it is not clear from methodology that it wasn’t randomised)  Unclear risk of bias? Y/N  Why? (don’t state it)  Low risk of bias? Y/N  Why? (state it) |
|  | Other comments: |  |
| ***Detection Bias*** | Were appropriate measures stated and used to detect changes in motor cortex corticospinal excitability? | High risk of bias? Y/N  Why? (used improper parameters)  Unclear risk of bias? Y/N  Why? (they haven’t given all the details)  Low risk of bias? Y/N  Why? (described everything really well and their methods were appropriate) |
|  | Were appropriate methods used to include only healthy participants (i.e., free of neurological or psychological impairments, free of pain)? | High risk of bias? Y/N  Why? (haven’t stated anything to say how they know they are healthy)  Unclear risk of bias? Y/N  Why? (not an established questionnaire or not given us info on what they have excluded)  Low risk of bias? Y/N  Why? (specifically states i.e. TMS screen questionnaire or some sort of checklist) |
|  | Other comments: | |
| ***Blinding of participants*** | Where possible, were participants blinded to the test condition or group they were in? | High risk of bias? Y/N  Why? (i.e. cover mirror and then use mirror makes it obvious to participant that the mirror is the active condition)  Unclear risk of bias? Y/N  Why? (they haven’t really said anything in manuscript and it is feasible that people couldn’t have known difference between conditions)  Low risk of bias? Y/N  Why? (state they were blinded i.e. sham tms or they may say they have blinded participants to the hypothesis of the study) |
|  | Other comments: | |
| ***Blinding of Researchers/outcome assessors***  *Who is involved (the person controlling TMS and the person talking to patient, who was blinded to what)* | Where able, was blinding between researchers of condition/outcome implemented? | High risk of bias? Y/N  Why? (if they haven’t said anything it should be high)  Unclear risk of bias? Y/N  Why? (one of the 2 has been blinded but still unsure of the person knowing)  Low risk of bias? Y/N  Why? (i.e. specifically stated they were blinded) |
|  | Other comments: | |
| ***Reporting Bias*** | Was there any evidence of selective outcome reporting? (e.g. were all outcomes and all groups reported on?)  *Check aims, methods and results for evidence*  Were outcome measures and results clearly presented? (i.e. can’t understand how they came to the conclusion that 2 groups were different and can’t see how results answer the question they asked) | High risk of bias? Y/N  Why? (talked about them in methods but didn’t report on all in results)  Unclear risk of bias? Y/N  Why? (not all data is reported on, not all participant data is reported on)  Low risk of bias? Y/N  Why? (all is reported on) |
|  | Other comments: | |
| ***Performance Bias***  ***(for illusion)*** | Was the effect of the illusion on body perception appropriately measured (e.g. embodiment questionnaire, proprioceptive drift) | High risk of bias? Y/N  Why? (if they haven’t used a measure or done it properly)  Unclear risk of bias? Y/N  Why? (if they said they done it but haven’t given details or details given we are not sure if it is valid i.e. embodiment is valid)  Low risk of bias? Y/N  Why? (if they report it and used it correctly) |
|  | Was the illusion appropriately described as well as the comparison condition? | High risk of bias? Y/N  Why? (unclear of how it happened or left out info)  Unclear risk of bias? Y/N  Why? (given info but impossible to interpret and is some missing info)  Low risk of bias? Y/N  Why? (they reported it well and we can replicate it) |
|  | Other comments: | |
| ***Confounding Variables*** | Were all confounding variables clearly stated (e.g. age, gender, hand dominance) and/or controlled for? (e.g. study design, matched controls, statistical analysis)  *for between subject study designs, confounding variables must not differ between groups or must be controlled for in the statistical analysis | High risk of bias? Y/N  Why? (not stated or missing or not controlled for. For within subject study designs if they have not mentioned about carry over effects it is high i.e. sufficient rest between conditions etc)  Unclear risk of bias? Y/N  Why? (they said they captured info but you can’t see how it is being controlled for in analyses)  Low risk of bias? Y/N  Why? (measured it and done it properly) |
|  | Other comments: | |
| ***Statistical Methods*** | Was an appropriate study design and size used with evidence of prior sample size calculation? | High risk of bias? Y/N  Why? (no sample size calc)  Unclear risk of bias? Y/N  Why? (they might say based on past studies but haven’t provided us with a calc)  Low risk of bias? Y/N  Why? (state a power calc) |
|  | Other comments: | |
| ***Attrition Bias*** | Was there any evidence of missing data? Was missing data less than 15% of the sample? | High risk of bias? Y/N  Why? (high risk if more than 15% missing data)  Unclear risk of bias? Y/N  Why? (not clear that the number of people at start are same in results. Check for each outcome can you tell all data is there, read results and figures)  Low risk of bias? Y/N  Why? (quite clear or they might say no missing data) |
|  | Other comments: | |

**Checklist for assessing the methodological quality of studies using TMS (Chipchase et al. 2012):**

| **Were the following participant factors** | **Reported?** | **Controlled?** |
| --- | --- | --- |
| Age of subjects | □ | □ |
| Gender of subjects | □ | N/A |
| Handedness of subjects | □ | □ |
| Subjects prescribed medication | □ | □ |
| Use of CNS active drugs (e.g. anti-convulsants) | □ | □ |
| Presence of neurological/psychiatric disorders when studying healthy subjects | □ | □ |
| Any medical conditions | □ | □ |
| History of specific repetitive motor activity | □ | □ |
| *Were the following methodological factors* | | |
| Position and contact of EMG electrodes | □ | □ |
| Amount of relaxation/contraction of target muscles | □ | □ |
| Prior motor activity of the muscle to be tested | □ | □ |
| Level of relaxation of muscles other than those being tested | N/A | □ |
| Coil type (size and geometry) | □ | □ |
| Coil orientation | □ | □ |
| Direction of induced current in the brain | □ | □ |
| Coil location and stability (with or without a neuronavigation system) | □ | □ |
| Type of stimulator used (e.g. brand) | □ | □ |
| Stimulation intensity | □ | □ |
| Pulse shape (monophasic or biphasic) | □ | □ |
| Determination of optimal hotspot | □ | □ |
| The time between MEP trials | □ | □ |
| Time between days of testing | □ | □ |
| Subject attention (level of arousal) during testing | □ | □ |
| Method for determining threshold (active/resting) | □ | □ |
| Number of MEP measures made | □ | □ |
| *Paired pulse only: Intensity of test pulse* | □ | □ |
| *Paired pulse only: Intensity of conditioning pulse* | □ | □ |
| *Paired pulse only: Inter-stimulus interval* | □ | □ |
| *Were the following analytical factors* |  |  |
| Method for determining MEP size during analysis | □ | □ |
| Size of unconditioned MEP | □ | □ |

Chipchase, L, Schabrun, S, Cohen, L, Hodges, P, Ridding, M, Rothwell, J, Taylor, J & Ziemann, U 2012, 'A checklist for assessing the methodological quality of studies using transcranial magnetic stimulation to study the motor system: an international consensus study', *Clinical Neurophysiology,* vol*.* 123, no. 9, pp. 1698-1704.
